# Multi-Pathway Cellular Analysis on Crude Natural Drugs/Herbs from Japanese Kampo Formulations

**DOI:** 10.1371/journal.pone.0128872

**Published:** 2015-06-02

**Authors:** Shizuka Eshima, Satoru Yokoyama, Takashi Abe, Yoshihiro Hayakawa, Ikuo Saiki

**Affiliations:** 1 Division of Pathogenic Biochemistry, Institute of Natural Medicine, University of Toyama, Toyama, Japan; 2 Graduate School of Science and Technology, Niigata University, Niigata, Japan; Hokkaido University, JAPAN

## Abstract

Kampo formulations comprise a number of crude natural drugs/herbs as constituents. The crude drugs/herbs have been traditionally classified by their traditional classifications or efficacies in Kampo medicines; however, it has been difficult to establish the scientific link between experimental evidence and traditional classifications in Kampo medicine. To clarify such traditional conceptions, we tested 112 crude drugs/herbs that are major components of Kampo formulations, in the multi-pathway analysis of 10 well-studied transcriptional activities including CREB, ERSF, HIF-1α, IRFs, MYC, NF-κB, p53, SMAD, SOX2, and TCF/LEF in A549 human lung cancer cells. By clustering the results of multi-pathway analysis with the Spearman rank-correlation coefficient and Ward linkage, three distinct traditional categories were significantly enriched in the major groupings, which are heat-clearing and dampness-drying herbs, acrid and warm exterior-resolving herbs, and acrid and cool exterior-resolving herbs. These results indicate that these crude drugs/herbs have similar effects on intracellular signaling and further imply that the traditional classifications of those enriched crude drugs/herbs can be supported by such experimental evidence. Collectively, our new *in vitro* multi-pathway analysis may be useful to clarify the mechanism of action of crude drugs/herbs and Kampo formulations.

## Introduction

Traditional Japanese (Kampo) medicine is based on traditional Chinese medicine but developed into a unique form through the accumulation of extensive experience and knowledge in Japan. Pathogenic alteration in Kampo medicine is based on the diagnosis of individual pathogenic alterations, so-called “Sho” in Japanese, comprising the symptoms and constitution (responder/non-responder) of patients with different diseases.

Over 100 Kampo formulations [1] that comprise some crude natural drugs/herbs as constituents have been approved as ethical drugs in the Japanese Pharmacopoeia (“Nihon Yakkyoku Ho” in Japanese), and clinically used for the treatment of a wide variety of diseases by physicians who are trained in Western medicine. Kampo formulations are classified into groups, such as heat-clearing, purgatives, and warming-interior, based on a treatment methodology corresponding to the “Sho” diagnosis [2]. In accordance with Kampo formulations, individual crude drugs/herbs are also similarly classified into major categories, such as heat-clearing and exterior resolving, and further sub-categories (heat-clearing and dampness-drying herbs, heat-clearing and toxin-resolving herbs) according to their definition of character, taste/nature, effects, and so on [2]. An approach involving therapies using Kampo medicines is highly impracticable, because Kampo medicines consist of multiple crude drugs and a large number of chemical substances. However, it is a significant challenge to predict the efficacy and mechanism of action of Kampo formulations and their crude drugs/herbs from the viewpoint of Western and modern medicine.

In order to examine the effects of 112 crude drugs/herbs on intracellular signaling comprehensively, we applied the reporter assay systems using synthetic plasmids with a cloned specific transcription factor-binding element upstream of the luciferase gene. By clustering analysis of the obtained results from such *in vitro* reporter assays, we found that three clusters of herb categories, namely, heat-clearing and dampness-drying herbs, acrid and warm exterior-resolving herbs, and acrid and cool exterior-resolving herbs, were significantly enriched in major groupings. The heat-clearing and dampness-drying herbs, such as Coptis Rhizome, Scutellaria Root, has some effects to clear heat and dry dampness An exterior-resolving medicine is known to induce sweating for expelling body surface factors cause illness, such as fever, algor, and head ache. Based on the experienced-based conceptions, there are two different exterior-resolving medicines, namely the acrid and warm exterior-resolving medicines or the acrid and cool exterior-resolving medicines, which increase or decrease body temperature, respectively. Considering those evidences and our presented results, the traditional categories of crude drugs/herbs in Kampo medicine may be positively associated with the effects on intracellular signaling using reporter assay unless there are significant differences in their botanical origin and active constituents. Alternatively, our results imply that the crude drugs/herbs belonging to the same category in Kampo medicine share a common effect on intracellular signaling activity *in vitro*. In summary, we propose that our comprehensive approach using reporter gene assay can be useful to predict the mechanism of action of Kampo formulations as well as crude drugs/herbs.

## Materials and Methods

### Reagents

Each crude drug/herb was added to an appropriate volume of distilled water (v/v, 1:8) and boiled at low flame for 50 min. The extracted solution was filtered and then freeze-dried to obtain dried powder. The voucher samples of these extracts (S1 Table) (INM_ID: 00000025–00000136, University of Toyama, http://shiryokannavi.inm.u-toyama.ac.jp/list#t1) were preserved in the Cooperative Research Project from Joint Usage/Research Center (Joint Usage/Research Center for Science-Based Natural Medicine), Institute of Natural Medicine, University of Toyama.

### Cell Cultures

A549 human lung cancer cells were obtained from American Type Culture Collection. The A549 cells were maintained in RPMI 1640 Medium (Life technologies, Carlsbad, CA, USA) containing 10% fetal bovine serum (FBS; ICN Biomedicals, Aurora, OH, USA), 1 mM L-glutamine (Life Technologies), 100 units/ml penicillin, and 100 μg/ml streptomycin in a humidified atmosphere of 95% air and 5% CO_2_ at 37°C.

### Plasmids

The tandem-repeated binding sites for ten transcription factors were subcloned into the pGL4.26 vector (Promega, Madison, WI, USA). The DNA oligonucleotides sequences subcloned are shown in S2 Table.

### Dual-luciferase assay

The A549 cells were co-transfected with each reporter plasmid and pRL-CMV *Renilla* control vector for 4–6 h using Lipofectamine2000 (Life Technologies) according to the manufacturer’s protocol, and replaced with fresh medium. The cells were further cultured with crude drugs/herbs (final 100 μg/ml) for 48 h and lysed with passive lysis buffer. The lysates were assayed by Dual-Luciferase Reporter Assay System (Promega). The reported results are the averages of two independent experiments, normalized for transfection efficiency using *Renilla* luciferase activity.

### Data analysis

Using relative transcriptional activities, the hierarchical clustering was performed by Spearman rank-correlation coefficient and Ward linkage with R statistical package (www.r-project.org). The results of hierarchical clustering were visualized as a heatmap. The heatmap was generated by plotting based on activity levels among the crude drugs/herbs using the program “heatmap.2” from gplots package in R. Activity levels were indicated by Z-scores calculated from the relative transcriptional activities of individual crude drugs/herbs across different reporters. The chi-square test was used to test for significant differences between the experience-based classifications of crude drugs/herbs.

### Real-time RT-PCR

Expression of *NR4A1 and HSP90B1* mRNA was quantitatively determined by real-time PCR on an ABI Prism 7300 sequence detection system (Life Technologies Corporation, Carlsbad, CA, USA). Total RNAs were prepared using the RNeasy Plus Mini kit (Qiagen, Hilden, Germany). Expression level of the targeted mRNAs was normalized to *β-actin* mRNA. The primers used were: 5’-ATC TTG GGA TTC TCC CTT CG-3’ (sense) and 5’-TCC CAT ATT GGG CTT GGA TA-3’ (antisense) for *NR4A1* mRNA, 5’-TGT AAT TGC TGA CCC AAG AGG-3’ (sense) and 5’-TCC AAT TCA AGG TAA TCA GAT GC-3’ (antisense) for *HSP90B1* mRNA, and 5’-GCA CAG AGC CTC GCC TT-3’ (sense) and 5’-GTT GTC GAC GAC GAG CG-3’ (antisense) for *ß-actin* mRNA.

## Results

**Fig 1:**
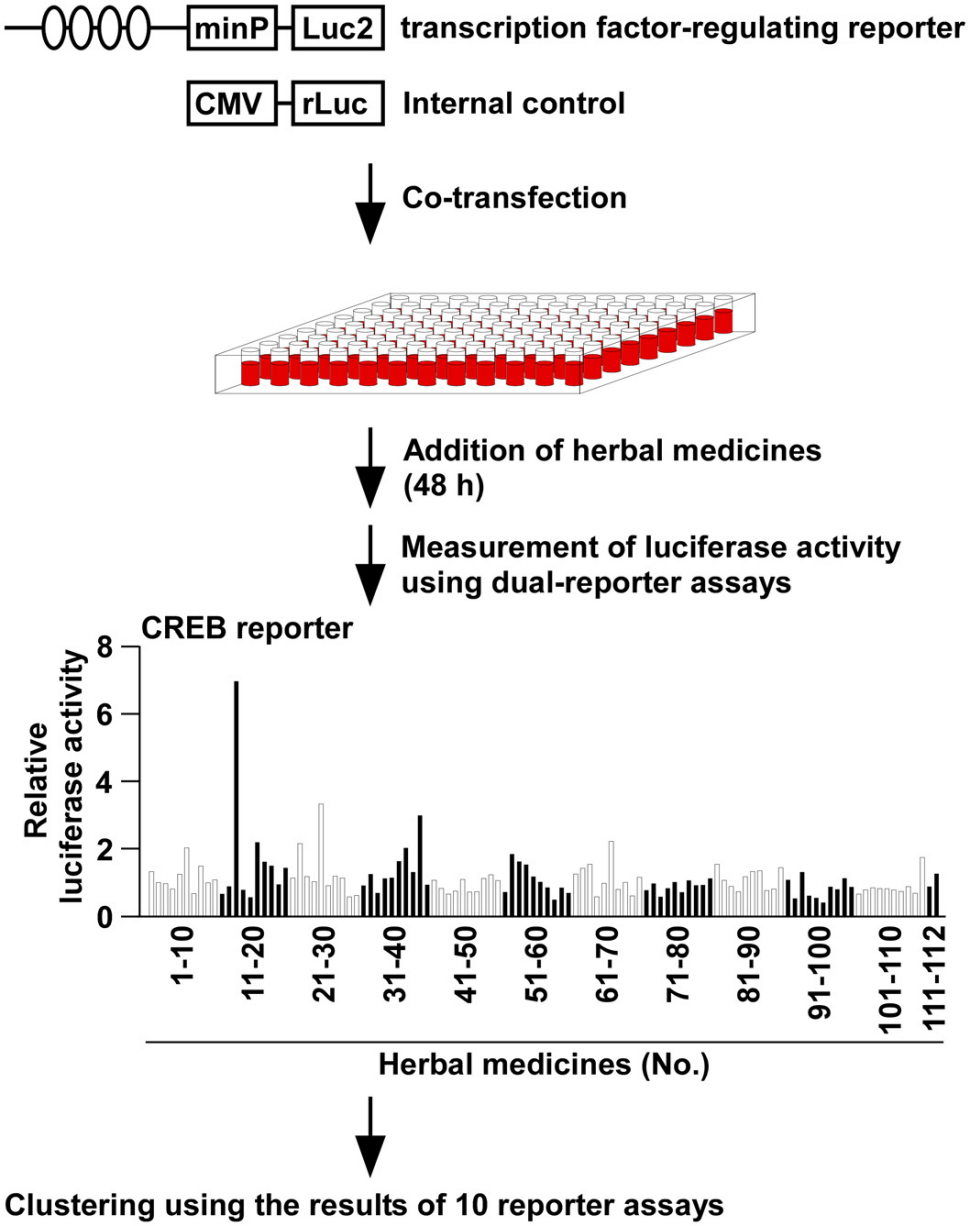
Schematic outline of the multi-pathway analysis using 112 extracts of crude drugs/herbs. A549 cells were co-transfected with the reporter plasmids containing the transcription factor-binding site upstream of the luciferase gene with the *Renilla* luciferase gene. After the addition of the extract of crude drugs/herbs at 100 μg/ml, the cell lysates were subjected to dual-luciferase assay. The luciferase activity of each crude drug/herb was normalized to that of the vehicle control. The result of CREB reporter was shown as an example. Data are mean of two independent experiments. The hierarchical clustering and heatmap were performed using the R statistical package.

**Fig 2:**
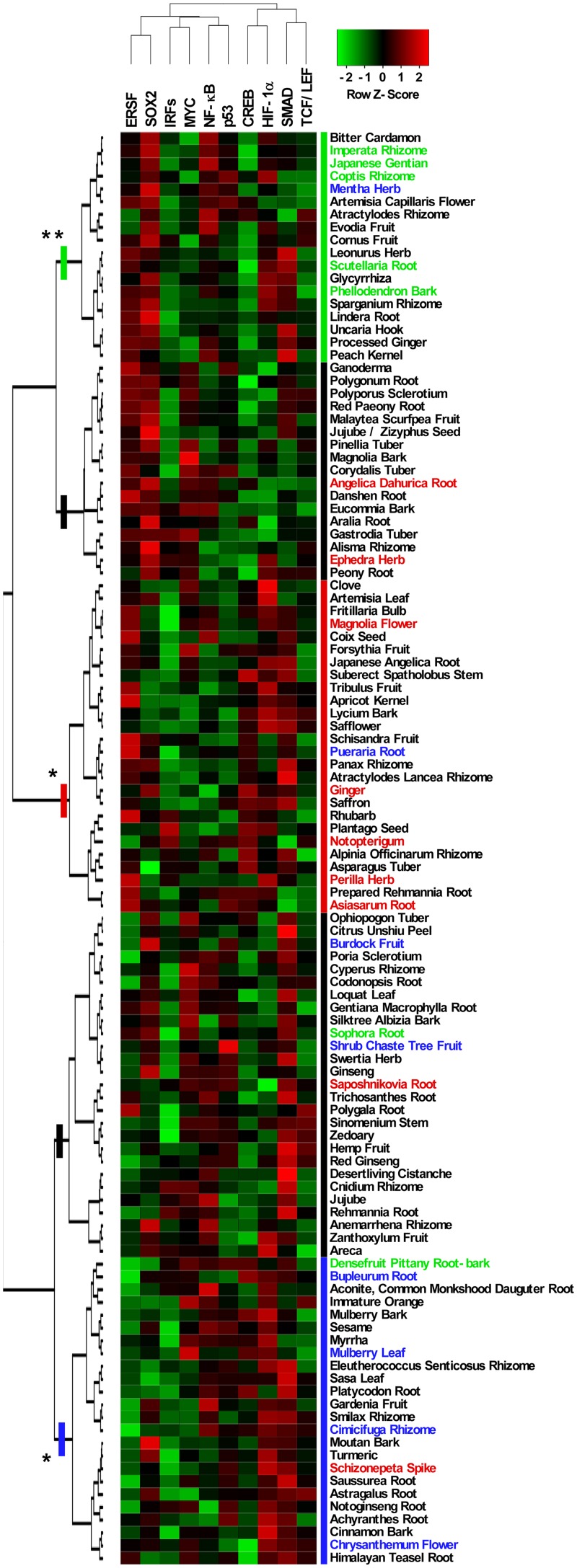
Panel of multi-pathway analysis of 112 crude drugs/herbs. Heatmap of activity levels of 112 crude drugs/herbs across 10 reporters. Activity levels were indicated by Z-scores calculated from the relative transcriptional activities of individual crude drugs/herbs across different reporters, and by colors presented at the top of the figure. The crude drugs/herbs belonging to three traditional conceptions (heat-clearing and dampness-drying herbs, the acrid and warm exterior-resolving herbs, and the acrid and cool exterior-resolving herbs) are shown in green, red, and blue, respectively. Five major groupings are shown as the lines on the tree of crude drugs/herbs, and the enriched major groupings of three traditional conceptions are shown in green, red, and blue, respectively. **P <0.01 and *P <0.*05* by chi-square test.

**Fig 3:**
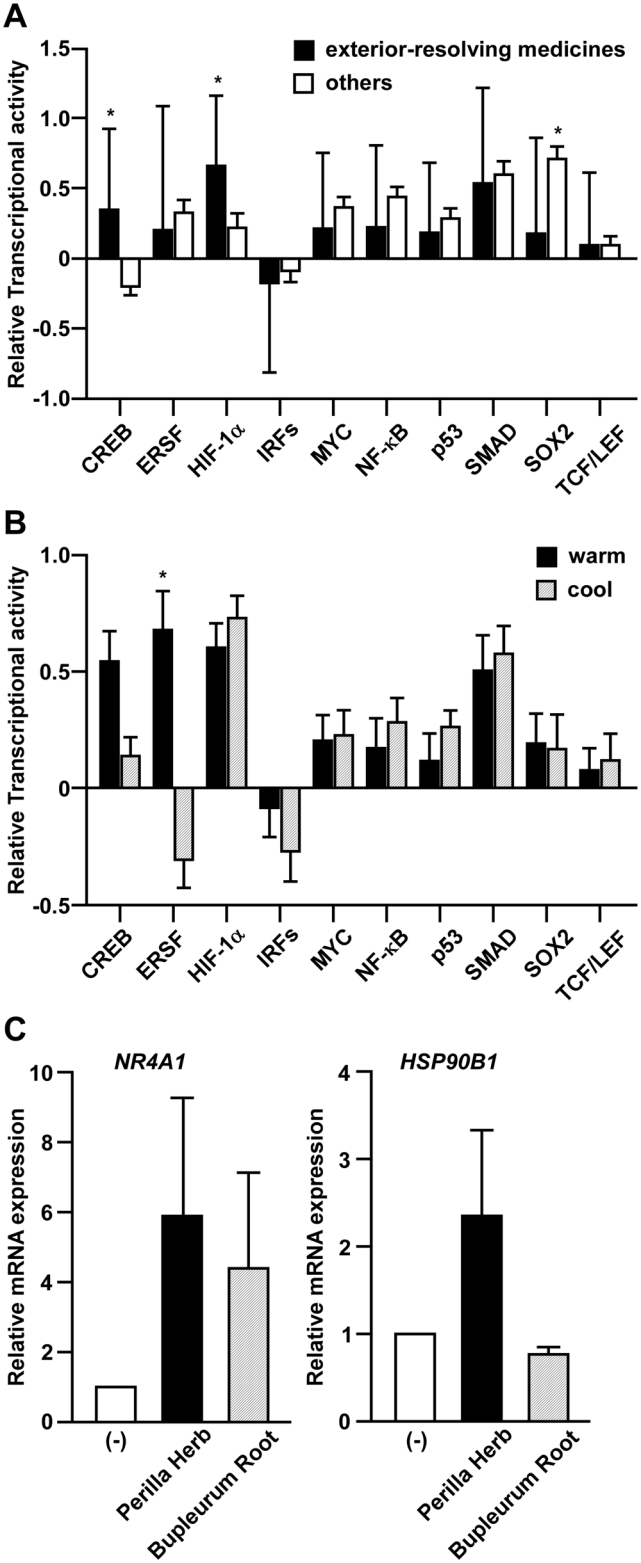
The different effects on each pathway by experiment-based classification. (A) Relative luciferase activity by each crude drug/herb was normalized to that of the vehicle control. The averages of relative luciferase activity of two major groupings containing exterior-resolving herbs (n = 50, filled bars) and others (n = 62, open bars) are shown. Data are the means ± S.D. of each grouping. *p <0.01 by two-way ANOVA followed by the Bonferroni *post-hoc* test compared with exterior-resolving medicines and others. (B) The averages of relative luciferase activity of each major groupings containing the acrid and warm exterior-resolving herbs (n = 24, filled bars) and the acrid and cool exterior-resolving herbs (n = 26, shadow bars) are shown. *p <0.01 by two-way ANOVA followed by the Bonferroni *post-hoc* test compared with warm and cool exterior-resolving medicines. Other conditions are similar to Fig 3A. (C) A549 cells were treated with vehicle (open bars), Perilla Herb (filled bars) or Bupleurum Root (shadow bars) for 48 h. NR4A1 (left panel) or HSP90B1 (right panel) was quantified by real-time RT-PCR. Relative mRNA expression was normalized to the value of each mRNA in vehicle-treated cells. Data are shown as the mean ± SD of three independent experiments.

Luciferase reporter assays are widely used to investigate intracellular signaling and used as high-throughput screening tools for drug discovery [3]. A collection of 112 crude drugs/herbs (S1 Table) were profiled along with their effects on 10 well-studied pathways, including cAMP response element binding protein (CREB), endoplasmic reticulum stress response factor (ERSF), hypoxia-inducible factor 1 alpha (HIF-1α), Interferon regulatory factors (IRFs), MYC, nuclear factor-kappa B (NF-κB), p53, SMAD, sex determining region Y-box 2 (SOX2) and T-cell transcription factor/lymphoid enhancer-binding factor (TCF/LEF) in A549 human lung cancer cells (Fig 1), because lung is one of the conceptive five viscera. The functional validation of these 10 reporters was conducted by using their known activators or inhibitors (data not shown). We then conducted the two-dimensional hierarchical clustering of relative transcriptional activities aligned with tested crude drugs/herbs to classify the 112 crude drugs/herbs into functionally similar groups by comparing the affected pathways. As summarized in Fig 2, the 112 crude drugs/herbs can be divided into five major groupings according to their effects on intracellular signaling; furthermore, and strikingly, three traditional Kampo classification, namely, heat-clearing and dampness-drying herbs, acrid and warm exterior-resolving herbs, and acrid and cool exterior-resolving herbs, were enriched in each major grouping, with statistical significance. Considering that two of those traditional classifications are related to exterior-resolving herbs, we next focused on the acrid and warm exterior-resolving herbs and the acrid and cool exterior-resolving herbs to subject them to further analysis. Among the 10 reporters tested, the effects on the CREB, HIF-1α, and SOX2 reporters were significantly different between the two groupings containing exterior-resolving herbs, compared to other groupings (Fig 3A). Given that CREB transcriptional activity was enhanced in the groupings containing exterior-resolving herbs but not in others, such transcriptional activation of CREB might be specific to exterior-resolving herbs and be related to the biological function or mechanism of action of exterior-resolving herbs. On the other hand, only ERSF transcriptional activity was significantly different between the two exterior-resolving herbs (Fig 3B), indicating that such a distinct effect on the ERSF pathway could explain the different biological efficacies between the acrid and warm exterior-resolving herbs and the acrid and cool exterior-resolving herbs. Consistent with the results of reporter assay, *NR4A1* mRNA, one of the CREB target genes [4], was induced by Perilla Herb or Bupleurum Root, which is an acrid and warm exterior-resolving herb or an acrid and cool exterior-resolving herb, respectively. We also detected the induction or the modest suppression of *HSP90B1* mRNA, known as an ERSF target gene [5], by Perilla Herb and Bupleurum Root.

## Discussion

In the present study, we subjected 112 crude drugs/herbs using 10 well-studied intracellular signaling panels using luciferase reporter assays, and divided the crude drugs/herbs into five major groupings. Among them, we identified that three traditional classifications (heat-clearing and dampness-drying herbs, acrid and warm exterior-resolving herbs, and acrid and cool exterior- resolving herbs) were significantly enriched in each major grouping.

Surprisingly, crude drugs/herbs can be classified into five groupings by using only 10 reporters in A549 cells (Fig 2). Although we recognize that our signaling panel with the 10 reporters in a single cell line is not sufficient to predict all of the intracellular events and *in vivo* efficacy of crude drugs/herbs, three traditional classifications were significantly enriched in those five major groupings of the 112 crude drugs/herbs. These studies using reporter assay support the usefulness of our *in vitro* classifications approach for predicting traditional Kampo categories with experimental evidence.

Crude drugs/herbs have been traditionally classified into about 22 minor categories by the definition of their character, effects, taste/nature, etc. We here demonstrated that only three classifications were significantly enriched among those 22 categories: heat-clearing and dampness-drying herbs, acrid and warm exterior-resolving herbs, and acrid and cool exterior- resolving herbs (Fig 2). This limitation may be due to our selected 10 intracellular signaling panels preferentially capturing the characteristics of these heat-related Kampo categories. Indeed, by focusing on the acrid and warm exterior-resolving herbs (Ginger, Magnolia Flower, Perilla Herb, etc), we found that CREB or ERSF transcriptional activation can be the characteristic pathways to the acrid and warm exterior-resolving herbs (Fig 3). Moreover, Kakkonto (in Japanese) or Ge-gen-tang (in Chinese), which is regarded as an acrid and warm exterior-resolving formulation, also showed CREB or ERSF transcriptional activation (data not shown), implying that our present new approach could be helpful to clarify the common mechanism of action between Kampo formulations and crude drugs/herbs as constituents.

In summary, we identified the functional similarities among 112 tested crude drugs/herbs based on their specific influences on intracellular signaling. This new *in vitro* approach could provide a new opportunity to clarify the mechanism of action of crude drugs/herbs and Kampo formulations. Considering that each drug/herb showed different activities on 10 reporters, such “fingerprints” of crude drugs/herbs would also be useful for the quality-control of Kampo medicines or the evaluation of authenticity of crude drugs/herbs.

## Supporting Information

S1 Table The list of crude drugs/herbs. (XLSX)

S2 Table The list of DNA oligonucleotide sequences. (XLSX)

## References

[1] Hijikata Y (2006) Analgesic treatment with Kampo prescription. Expert Rev Neurother 6: 795–802. 16734526 10.1586/14737175.6.5.795

[2] Yz S, Igakkai NT. (2005) Introduction to KAMPO: Japanese Traditional Medicine Tokyo: Elsevier Japan xvi, 286 p. p.

[3] Brasier AR, Ron D (1992) Luciferase reporter gene assay in mammalian cells. Methods Enzymol 216: 386–397. 1336098 10.1016/0076-6879(92)16036-j

[4] Fallahi M, Amelio AL, Cleveland JL, Rounbehler RJ (2014) CREB targets define the gene expression signature of malignancies having reduced levels of the tumor suppressor tristetraprolin. PLoS One 9: e115517 doi: 10.1371/journal.pone.0115517 25541715 10.1371/journal.pone.0115517 PMC4277357

[5] Hetz C (2012) The unfolded protein response: controlling cell fate decisions under ER stress and beyond. Nat Rev Mol Cell Biol 13: 89–102. doi: 10.1038/nrm3270 22251901 10.1038/nrm3270

